# Biodegradable Mg-Sc-Sr Alloy Improves Osteogenesis and Angiogenesis to Accelerate Bone Defect Restoration

**DOI:** 10.3390/jfb13040261

**Published:** 2022-11-22

**Authors:** Nadia Aboutalebianaraki, Craig J. Neal, Sudipta Seal, Mehdi Razavi

**Affiliations:** 1 Biionix^TM^ (Bionic Materials, Implants & Interfaces) Cluster, Department of Medicine, University of Central Florida College of Medicine, Orlando, FL 32827, USA; 2 Department of Materials Science and Engineering, University of Central Florida, Orlando, FL 32816, USA; 3 Advanced Materials Processing and Analysis Center, Department of Materials Science and Engineering, University of Central Florida, Orlando, FL 32826, USA

**Keywords:** magnesium alloy, osteogenesis, angiogenesis, bone implants

## Abstract

Magnesium (Mg) and its alloys are considered to be biodegradable metallic biomaterials for potential orthopedic implants. While the osteogenic properties of Mg alloys have been widely studied, few reports focused on developing a bifunctional Mg implant with osteogenic and angiogenic properties. Herein, a Mg-Sc-Sr alloy was developed, and this alloy’s angiogenesis and osteogenesis effects were evaluated in vitro for the first time. X-ray Fluorescence (XRF), X-ray diffraction (XRD), and metallography images were used to evaluate the microstructure of the developed Mg-Sc-Sr alloy. Human umbilical vein/vascular endothelial cells (HUVECs) were used to evaluate the angiogenic character of the prepared Mg-Sc-Sr alloy. A mix of human bone-marrow-derived mesenchymal stromal cells (hBM-MSCs) and HUVEC cell cultures were used to assess the osteogenesis-stimulating effect of Mg-Sc-Sr alloy through alkaline phosphatase (ALP) and Von Kossa staining. Higher ALP activity and the number of calcified nodules (27% increase) were obtained for the Mg-Sc-Sr-treated groups compared to Mg-treated groups. In addition, higher VEGF expression (45.5% increase), tube length (80.8% increase), and number of meshes (37.9% increase) were observed. The Mg-Sc-Sr alloy showed significantly higher angiogenesis and osteogenic differentiation than pure Mg and the control group, suggesting such a composition as a promising candidate in bone implants.

## 1. Introduction

The growing number of musculoskeletal disorders caused by poorly healed fractures and diseases has become a serious global challenge in public health, significantly increasing overall healthcare costs for patients [1,2,3,4,5]. Recently, permanent metallic biomaterials, including titanium alloys, stainless steels, and cobalt-chromium alloys, have been used to fix bone fractures [5,6,7]. These metallic implants can only provide physical support at the fracture; they do not contribute to bone regeneration [5,6,7]. Further, concerns have been raised over the deleterious medical effects of mechanical property mismatches between such materials and cortical bones. Differences in elastic modulus between the implant and natural bone lead to unnatural, inhomogeneous mechanical loading, resulting in implant loosening, reduction in osteogenesis, implant failure, and removal surgery [1,7]. Meanwhile, the presence of these permanent implants in the fractured site after the complete healing of the bone can result in the release of toxic degradation-related particles and ions in the long term. This may cause chronic inflammation by triggering a host immune response, leading to tissue damage and implant failure [5,8].

Mg is a biodegradable metal that eliminates the need for removal surgery [9,10]. It has a Young’s modulus (10–40 GPa) close to that of human cortical bone (3–20 GPa) [11,12]. More importantly, Mg^2+^, which evolves from the implant over time post-implantation, is one of the most predominant elements in the human body, mainly in bones and teeth [12]. Mg ions can participate in numerous bone-related biological functions and processes related to cell metabolism [13,14]. Mg^2+^ can improve new bone formation, regulate the resorption process, upregulate the expression of osteogenic genes, increase the mineralization of Ca^2+^, promote osteoblast adhesion to the implant surface, and regulate muscle contraction [14,15,16].

Angiogenesis is a critical factor in facilitating new bone formation by mediating the mass transport of sufficient oxygen and nutrients through new vessel formation in the early healing process and increasing the success rate of implantation [9,17,18]. Mg has also been found to enhance the extent and rate of angiogenesis at implant sites [18,19]. Additionally, Mg^2+^ induces nitric oxide production by endothelial cells, which upregulates the vascular endothelial growth factor (VEGF) expression and improves angiogenesis [9,20,21]. These characteristics indicate the excellent potential of Mg as a biomaterial in bone healing applications [5,9,22].

Although previous investigations have demonstrated the potential of Mg alloys in bone defect restoration, the high corrosion rate of Mg implants leads to the generation of substantial amounts of subcutaneous hydrogen (H_2_) gas at the implantation site. The gas formation reduces implant mechanical integrity, resulting in damage to the surrounding tissues and implant failure [5,10,15]. Mg alloy’s highly alkaline local pH value can also hinder the healing process since a high pH would damage cells [23]. Therefore, controlling the degradation rate and resultant H_2_ generation of Mg implants is crucial for the successful bone healing process, limitation of tissue damage, and maintenance of a healthy tissue-implant microenvironment [8,9]. Recently, several alloying elements frequently used with Mg, such as zirconium or aluminum, were found to produce unfavorable effects on tissue health (i.e., toxicity induction) [24]. Suitable alloying components for bone implants should be biocompatible and enhance biomechanical support, osseointegration, osteogenesis, and angiogenesis [9,21,25].

Aiming to reduce the corrosion rate of Mg, we have used scandium (Sc) as an alloying element in Mg due to its biocompatibility, high maximum solubility limit (24.61 wt.%), and similar lattice parameters to Mg [2,26] (which should significantly decrease lattice distortion and, thereby, effectively hinder stress concentration). Incorporating bioactive elements such as Sr, F, and Zn in Mg alloys has gained interest recently [2,26,27]. Among these bioactive elements, Sr, due to its similar structure to Ca, can provide the additional advantage of inducing osteoprogenitor cell differentiation into bone-forming osteoblasts by activating the Wnt/β-catenin signaling pathway and calcium-sensing receptors (CaSR). This also reduces the bone resorption that, in conjunction with the previously detailed processes, leads to superior new bone formation [5,8,14,28,29]. Sr ions can also bind to the bone matrix, increase bone mineralization [10,29], and enhance angiogenic properties [27,28,30].

The Mg-Zr-Sr-Sc alloy was developed based on a previous report [2]. However, this is the first time that Mg-Sc-Sr was developed, and the mechanism of bone formation using ALP and Von Kossa staining and vascularization using wound healing were studied. Finally, for the first time, this study offers a tube formation assay conducted on the Mg-Sc-Sr alloy. 

To integrate osteogenesis and angiogenesis, we developed an Mg alloy containing Sc and Sr (Mg-Sc-Sr alloy). We hypothesized that the Sc component would improve corrosion resistance, while Sr addition would enhance/induce bone-forming processes. XRD, XRF, and metallography were performed to analyze the Mg-Sc-Sr alloy microstructure. An evaluation of early angiogenesis stages in human umbilical vein/vascular endothelial cells (HUVECs) was performed by vascular endothelial growth factor induction using an enzyme-linked immunosorbent assay (VEGF ELISA), tube formation, and scratch tests.

Moreover, in this work, human bone marrow-derived mesenchymal stromal cells (hBM-MSCs) and HUVECs were co-cultured to study the in vitro osteogenesis effects of the Mg-Sc-Sr alloy implant in a similar environment to bone tissue. The osteogenesis character of Mg-Sc-Sr alloy was examined by analyzing the Alkaline phosphatase (ALP) activity and Von Kossa staining.

## 2. Materials and Methods

### 2.1. Mg Alloy Fabrication

The Mg (as control) and Mg-Sc-Sr alloys (2 wt.% Sc, and 2 wt.% Sr) were manufactured by casting method using high purity Mg (Goodfellow, Pittsburgh, PA, USA), Sc (Luciteria science, Olympia, WA, USA), and Sr (Luciteria science, Olympia, WA, USA). The Mg, Sc, and Sr were melted at 750 °C in a graphite crucible. After 10 min, the melted alloy was cast in a cylindrical mold. The melt was protected by an inert high-purity argon (Ar) atmosphere. Next, the Mg-Sc-Sr rods were heat-treated at 400 °C for 2 h. They were then cut into 12 mm diameter and 5 mm height disks for microstructure characterizations, osteogenesis, and angiogenesis evaluation as described below. Finally, discs were wet ground with successive grades of silicon carbide (SiC) abrasive paper (400, 600, 1200 grit) and ultrasonically cleaned (50 Hz and 150 W) in ethanol for 15 min and air-dried for further analysis. 

### 2.2. Microstructure Characterization 

The phase constitutions of the Mg and Mg-Sc-Sr disks were detected by X-ray diffraction (XRD) using an X-ray diffractometer (PANalytical Empyrean, Netherlands) with Cu-K*α* radiation (*λ* = 0.154 nm, 40 kV) at a step size of 0.02°/s over the angular range of 10–90° (2θ value). The international center of diffraction data (ICDD) PDF-4 database was used for phase identification. The elemental phase compositions of the Mg and Mg-Sc-Sr disks were detected by X-ray Fluorescence (XRF) using an X-ray fluorescence spectrometer (PANalytical Epsilion, Netherlands) with a 50 KV silver anode. 

The surface chemistry and chemical compositions were examined using X-ray photoelectron spectroscopy (XPS, Physical electronics PHI 5802). A non-monochromatic aluminum K*α* X-ray source (51,486.6 eV, 250 W) with an acceleration voltage of 20 kV and amperage of 15 mA was used for all measurements. Measurements were performed under vacuum at <10^−8^ mbar. Core-level XPS spectra for C1s, Mg2p, and O1s were recorded. The binding energy values were referenced to a C1s peak (hydrocarbon C–C, C–H) at 284.6 eV, ascribed to adventitious carbon. A smart (Thermo Avantage v5.9902, Thermo Fisher) fitted background was applied to all collected spectra.

To observe sample microstructure and grain character, a picric acid-based etchant composed of 50 mL ethanol (100% *v*/*v*, Decon Labs, Montgomery, PA, USA), 5 mL acetic acid (Fisher, Pittsburgh, PA, USA), and 3 mL picric acid (LabChem, Zelienople, PA, USA) was used for etching the discs. 

Finally, the microstructure was observed using an optical microscope (Amscope FMA-50, Irvine, CA, USA), and the line intercept method (ASTM, 2013) was used to calculate the average grain sizes for each disc [31].

### 2.3. Angiogenesis

Human umbilical vein/vascular endothelial cells (HUVECs) were obtained from American Type Culture Collection (ATCC, PCS-100-010, Manassas, WA, USA). HUVECs were cultured in a complete endothelial cell growth medium containing Vascular Cell Basal Medium (ATCC, PCS-100-030, Manassas, WA, USA) supplemented with Endothelial Cell Growth Kit-BBE (ATCC, PCS-100-040, Manassas, WA, USA) and incubated in a humidified atmosphere under the condition of Atm (atmospheric) O_2_ and 5% CO_2_ at 37 °C and incubated until cells reached to 80–90% confluency. To prepare endothelial cells extract, Mg and Mg-Sc-Sr disks were immersed in a complete endothelial cell growth medium (disc weight/medium volume ratio was 0.2 g/mL) [11]. The tube formation assay was performed as described before [9,11]. 

Briefly, a 96-well plate was coated with 50 µL of ice-cold Matrigel in each well (BD Bioscience, San Jose, CA, USA) on the ice. Then, the well plate was kept in the humidified atmosphere under the condition of Atm O_2_ and 5% CO_2_ for 30 min at 37 °C to allow gelation of the Matrigel. Next, the HUVECs (3 × 10^4^/well) were seeded onto Matrigel-coated wells and cultured in a complete endothelial cell growth medium. Cells were incubated in a humidified atmosphere under Atm O_2_ and 5% CO_2_ for 4 h, 24 h, and 48 h at 37 °C after adding 200 µL of Mg and Mg-Sc-Sr extract. HUVECs were cultured with a complete endothelial cell growth medium without an extraction medium as a control. 

Cells were then imaged at each time point using an optical microscope. The ability of HUVECs to form tubes was quantified by measuring the sum of tube length in each image using ImageJ software and reported as total tube length. Also, the total number of completed circular meshes in each image was counted and reported as the number of meshes. Five images per experimental group were analyzed. 

To study the wound healing ability of Mg-Sc-Sr alloy, HUVECs (5 × 10^5^/well) were seeded in six-well plates and cultured with a complete endothelial cell growth medium in a humidified atmosphere under the condition of Atm O_2_ and 5% CO_2_ at 37 °C for 24 h. A straight line was scratched on the confluent monolayer the next day by scraping with sterile 1000 µL pipette tips. Cells were then rinsed three times with PBS, and the prepared Mg and Mg-Sc-Sr alloy extracts were added to each well (1000 µL/well). As controls, HUVECs were cultured with a complete endothelial cell growth medium. Finally, cells were imaged after 4 h, 24 h, and 48 h using an optical microscope. To quantify the wound healing ability and cell migration activities of HUVECs, the number of migrated cells and scratch width were measured for five images per experimental group. The scratch width was measured using ImageJ software (version 1.53k).

To measure the VEGF expression from HUVECs, cells (3 × 10^4^/well) were seeded in 96-well plates and cultured with a complete endothelial cell growth medium in a humidified atmosphere under the condition of Atm O_2_ and 5% CO_2_ at 37 °C for 24 h. Then, 100 µL Mg or Mg-Sc-Sr alloy extracts were added to each well. The HUVECs cultured with a complete endothelial cell growth medium were considered as control. The concentration of VEGF in the supernatant of HUVECs for each group was measured using a Human VEGF ELISA Kit (Boster, Pleasanton, CA, USA) after 4 h, 10 h, and 24 h of incubation, according to the manufacturer’s instruction. Briefly, 100 µL supernatant of each experimental group was added to each well of the ELISA kit well plate. Then, the well plate was covered with the plate sealer and incubated for 90 min at 37 °C. Next, 100 µL of the Biotinylated Anti-Human VEGFA antibody was added to each well, and the well plate was incubated for 60 min at 37 °C. Afterward, the wells were washed with wash buffer, 100 µL of the Avidin-Biotin-Peroxidase Complex was added to each well, and the well plate was incubated at 37 °C for 30 min. Finally, 90 µL of Color Developing Reagent was added to each well for 15 min at 37 °C, and the reaction stopped by adding 100 µL of Stop Solution. The optical density of each well was determined by using a microplate reader (Bio-Rad 680, Hercules, CA, USA) at 450 nm.

### 2.4. Osteogenesis 

Human bone marrow-derived mesenchymal stromal cells (hBM-MSCs, ATCC, PCS-500-012, Gaithersburg, MD, USA) were cultured in a complete cell culture medium composing Low glucose Dulbecco’s Modified Eagle’s medium (DMEM; glucose concentration: 1 g/L, Invitrogen, Carlsbad, CA, USA) supplemented with 50 U/mL penicillin-50 µg/mL streptomycin (Lonza, Basel, Switzerland) and 10% fetal bovine serum (FBS; Invitrogen, Carlsbad, CA, USA) at 37 °C under the condition of Atm O_2_ and 5% CO_2_ in a humidified atmosphere. The cell culture medium was refreshed every three days until cells reached 80–90% confluency. Then, to prepare Mg and Mg-Sc-Sr differentiation extracts, disks were first sterilized with ethanol, washed three times with sterile PBS, and sterilized with ultraviolet light for 2 h. Next, the discs were immersed in a differentiation medium (complete cell culture medium supplemented with 10 mM β-glycerophosphate, 50 mM ascorbic acid, and 100 nM dexamethasone (Sigma-Aldrich, Burlington, MA, USA)) [32] and incubated for three days in a humidified atmosphere at 37 °C under the condition of Atm O_2_ and 5% CO_2_ to obtain extract [33]. The discs’ weight/medium volume ratio was 0.2 g/mL [33]. The acquired extracts of each disc were centrifuged, the solution was collected and stored separately at 4 °C, and the collected differentiation extracts were then used for Von Kossa staining and ALP staining.

To perform ALP staining and Von Kossa staining, hBM-MSCs (5 × 10^4^ cells/well) and HUVECs (5 × 10^4^ cells/well) were mixed and seeded in 12-well plates and cultured in a 1:1 mix of the complete cell growth medium and complete endothelial cell growth medium for 1 day at 37 °C in a humidified atmosphere under the normal condition of Atm O_2_ and 5% CO_2_. Then, the culture medium was replaced and hBM-MSCs-HUVECs were cultured (7 days for ALP staining and 21 days for Von Kossa staining) with a 1:1 mix of differentiation extract and endothelial cell extract for Mg and Mg-Sc-Sr groups. The medium was refreshed every 3 days. The mix of HUVECs and hBM-MSCs cultured with a 1:1 mix of differentiation medium and endothelial cell growth medium with no sample extract is considered a control.

The ALP staining was performed using Alkaline Phosphatase Staining Kit (Abcam, Boston, MA, USA). On day 7, the hBM-MSCs and HUVECs were washed with wash buffer. Then, 150 µL of ALP Staining Reagent solution was added to each well and incubated at 37 °C for 20 min. Finally, the cells were imaged using BZ-X810 Keyence. 

Von Kossa staining was performed using a Von Kossa Stain Kit (Stablab, McKinney, TX, USA). On day 21, the medium was removed, hBM-MSCs and HUVECs were rinsed with DI water, and a 5% silver nitrate solution was added to each well. Then, cells were irradiated with ultraviolet light for 15 min. Once completed, cells were rinsed in DI water, and the 5% sodium thiosulfate solution was added for 1 min. Finally, cells were incubated in the nuclear fast red stain for 5 min, washed, and imaged (BZ-X810, Keyence, Itasca, IL, USA). To quantify the Von Kossa staining images, the number of black precipitates was counted as the number of nodules in five images per group using ImageJ software (version 1.53k) [34]. 

### 2.5. Statistical Analysis

Metallography, osteogenesis, and angiogenesis were performed in *n* = 5 samples per group. The XRD and XPF were performed in *n* = 1, and XPS was done in *n* = 3 samples per group. Results were reported as mean ± standard deviation. One-way ANOVA followed by a post-hoc Tukey test (Astatsa.com, Online and Free Web Statistical Calculators) and two-way ANOVA using GraphPad Prism software were used to obtain statistical significance between groups. The *p*-value of <0.05 denotes a statistically significant result.

## 3. Results

### 3.1. Microstructure Characterization 

**Figure 1:**
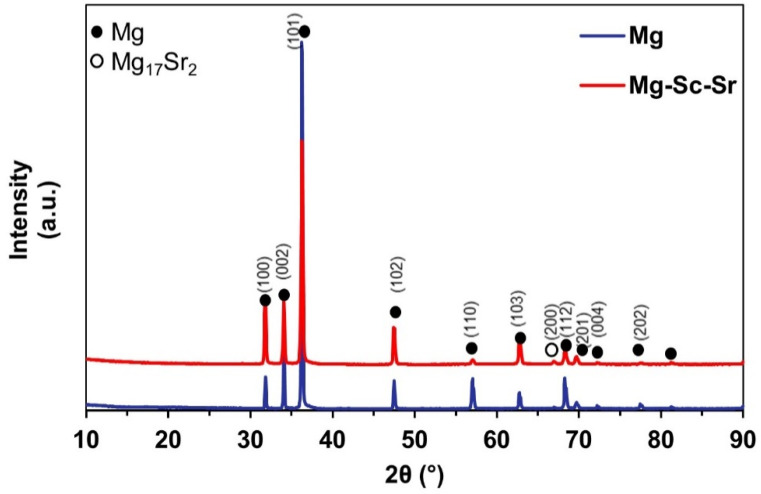
XRD patterns of the Mg and Mg-Sc-Sr alloy showing α-Mg phase for Mg sample and α-Mg and Mg_17_Sr_2_ intermetallic phase for Mg-Sc-Sr alloy.

The chemical compositions of Mg and Mg-Sc-Sr alloys are presented in Table 1. The phase analysis of Mg and Mg-Sc-Sr discs was evaluated using X-ray diffraction (XRD). Figure 1 shows the XRD pattern of Mg that includes an α-Mg phase with diffraction peaks at 32.17°, 34.4°, 36.59°, 47.78°, 57.30°, 63.03°, 68.57°, 69.93°, 72.44°, 77.75°, and 81.09° (JCPDS No. 35-0821). Also, as shown in Figure 1, the Mg-Sc-Sr pattern indicated the α-Mg phase and Mg_17_Sr_2_ intermetallic phase at 67.25°, representing the formation of Mg_17_Sr_2_ in Mg-Sc-Sr discs (JCPDS No. 18-1275) [35]. Due to the high dissolution of Sc in Mg (24.61 wt.%), Sc element is well-dispersed in Mg, and no separate peaks were detected. As Figure 1 shows, adding more than 0.11 wt.% of the Sr (e.g., 2 wt.%) as an alloying element in Mg resulted in the alloying of some fraction of Sr content into the Mg, with the remainder contributing to the formation of an intermetallic phase (Mg_17_Sr_2_) [31].

**Figure 2:**
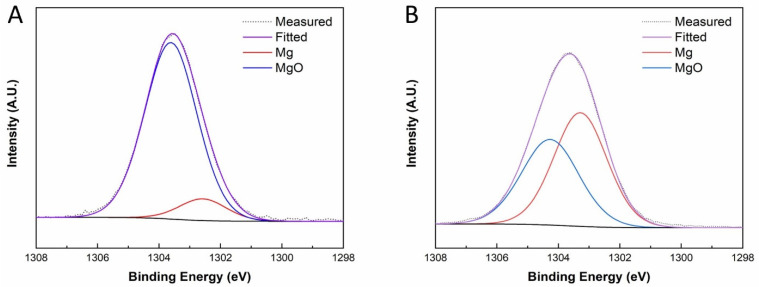
XPS spectra: (**A**) Mg 1s for Mg sample, (**B**) Mg 1s for Mg-Sc-Sr sample. We observed a lower oxide proportion relative to the Mg-Sc-Sr sample’s metallic state.

XPS analysis was performed to evaluate the chemical composition of the surface layers on Mg and Mg-Sc-Sr discs. As shown in the de-convoluted XPS spectra (Figure 2A,B), peaks fitted in the binding energy range specific to Mg (1s) were ascribed to metallic (Mg^0^) and oxide (Mg^2+^) chemical states for both Mg sample (Figure 2A) and Mg-Sc-Sr sample (Figure 2B). The presence of both states is typical of a material surface wherein Mg is a major phase (or parent material), given the material’s negative reduction potential. In the case of the Mg-Sc-Sr alloy sample, the ratio of fitted peak areas (measurement intensity area under-the-curve values) for Mg^0^:Mg^2+^ was lower (0.8) compared to the pure Mg (2.5). Along with this, a shift in binding energy was observed to higher values, for both Mg1s-associated peaks, in the alloyed sample (+Δ0.4 and 0.6 eV, for Mg^0^ and Mg^2+^, respectively). This shift thereby suggests greater stability of related chemical states at the surface of the Mg-Sc-Sr alloy sample.

**Figure 3:**
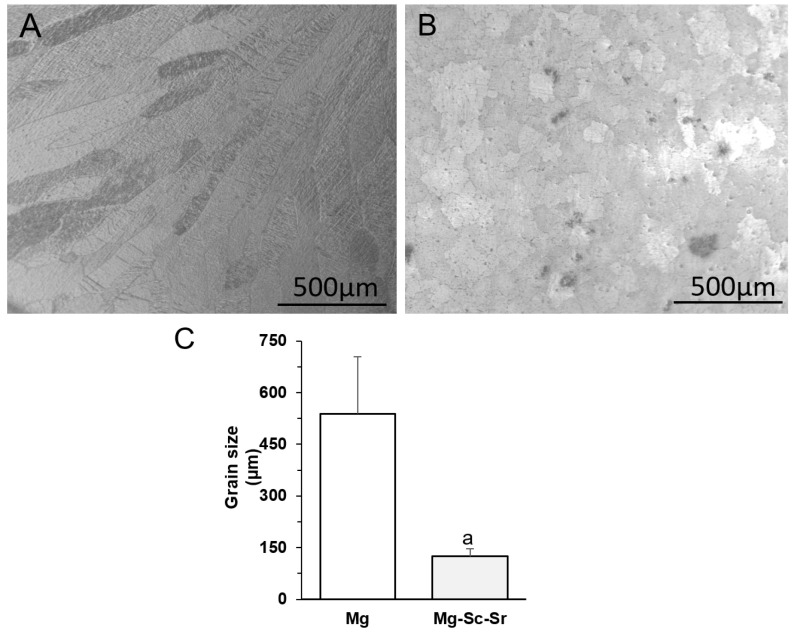
Metallography images: (**A**) Mg, (**B**) Mg-Sc-Sr, (**C**) average grain sizes of the Mg and Mg-Sc-Sr discs. In comparing the Mg-Sc-Sr grain size to the Mg grain size, a significant reduction in grain size for Mg-Sc-Sr was observed. Significant differences: a: *p* < 0.05 Mg-Sc-Sr vs. Mg.

Figure 3A–C showed that the average grain size for Mg-Sc-Sr (124.86 ± 22.61 µm) was significantly lower compared to Mg (539.16 ± 164.58 µm) due to the grain refinement effect of Sc [36,37,38] and Sr [35,39,40]. Intermetallic particles in the Mg-Sc-Sr discs could also cause a reduction in grain size through the grain-growth restriction mechanism [31,41,42].

### 3.2. Angiogenesis

**Figure 4:**
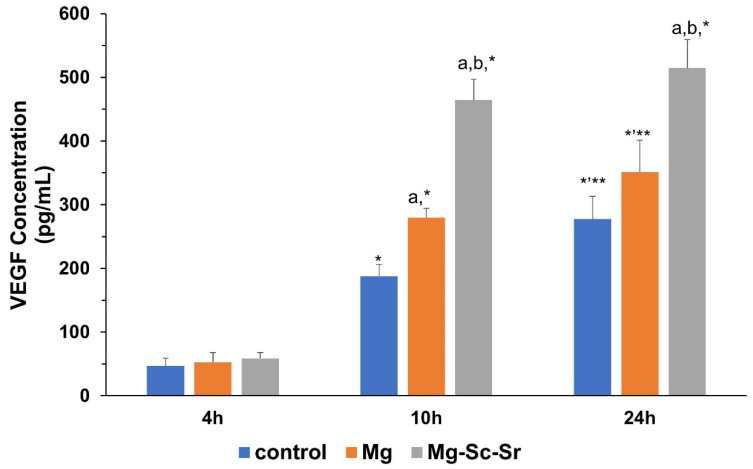
VEGF expression from HUVECs after 4 h, 10 h, and 24 h culturing with the control, Mg, and Mg-Sc-Sr extracts in vitro showing a significant increase in VEGF concentration for Mg-Sc-Sr-treated HUVECs compared to control. Significant differences based on two-way A: a: *p* < 0.05 Mg and Mg-Sc-Sr vs. control, b: *p* < 0.05 Mg-Sc-Sr vs. Mg. *: *p* < 0.05 24 h and 10 h vs. 4 h, **: *p* < 0.05 24 h vs. 10 h for each experimental group.

To detect the expression of VEGF secreted from HUVECs cultured alone as control or HUVECs cultured with Mg, and Mg-Sc-Sr extracts, a VEGF ELISA was performed after 4 h, 10 h, and 24 h of incubation (Figure 4). After 4 h of incubation, the VEGF concentration of HUVECs cultured with Mg-Sc-Sr extract (58.83 ± 8.89 pg/mL) and Mg (52.66 ± 15.22 pg/mL) showed insignificant changes (*p* > 0.05) compared to VEGF concentration in the control group (46.93 ± 11.96 pg/mL). After 10 h of incubating HUVECs with the control, Mg, and Mg-Sc-Sr extract, the VEGF concentration for Mg (279.53 ± 14.66 pg/mL) and Mg-Sc-Sr (464.55 ± 32.31 pg/mL) obtained significantly higher than the control (187.90 ± 18.36 pg/mL, *p* < 0.05). The measured VEGF concentration of the HUVECs cultured with Mg-Sc-Sr was higher than the VEGF concentration of HUVECs cultured with Mg (*p* < 0.05). A significantly higher concentration of VEGF in the supernatant of HUVECs cultured with Mg-Sc-Sr extract (514.33 ± 44.90 pg/mL) compared to Mg (350.90 ± 50.25 pg/mL) and control (270.33 ± 35.61 pg/mL) after 24 h of incubation indicated that Mg-Sc-Sr extract potentiates VEGF secretion. These results also showed that the VEGF concentration in all groups increased over time, and the Mg-Sc-Sr alloy has a higher pro-angiogenic potential relative to pure Mg.

In Mg extract-treated samples, the VEGF concentration at 10 h and 24 h of incubation were significantly higher than the VEGF concentration at 4 h, and the highest concentration of VEGF was obtained after 24 h. Similarly, for HUVECs cultured with Mg-Sc-Sr extract, the VEGF concentration at 10 h and 24 h of incubation was significantly higher than the VEGF concentration at 4 h (*p* < 0.05). However, no significant changes (*p* > 0.05) were observed between 10 h and 24 h.

**Figure 5:**
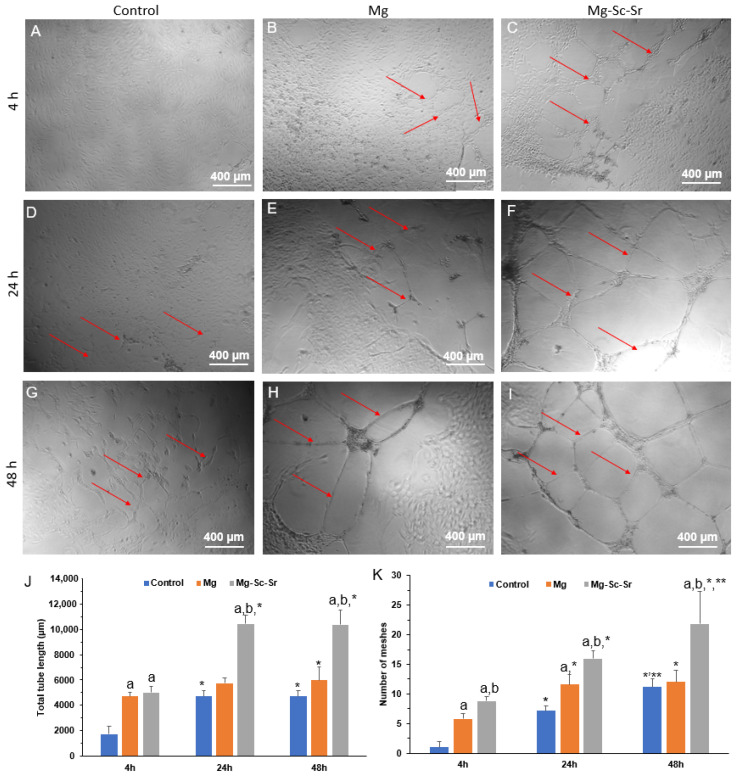
Tube formation assay for HUVECs cultured with the control, Mg, and Mg-Sc-Sr extracts in vitro. Tube formation images of HUVECs after culturing with the control group, Mg extract, and Mg-Sc-Sr extract after (**A**–**C**) 4 h, (**D**–**F**) 24 h, (**G**–**I**) 48 h, (**J**) quantification of total tube length formed by HUVECs, (**K**) quantification of the number of formed meshes by HUVECs, for control, Mg, and Mg-Sc-Sr at 4 h, 24 h, and 48 h. Significant differences based on two-way Anova: a: *p* < 0.05 Mg and Mg-Sc-Sr vs. control, b: *p* < 0.05 Mg-Sc-Sr vs. Mg, *: *p* < 0.05 48 h and 24 h vs. 4 h, **: *p* < 0.05 48 h vs. 24 h for each experimental group. Red arrows show newly formed tubes.

As shown in Figure 5A–C,J, the quantified total tube length for HUVECs cultured in the Mg extract (4708.8 ± 422.3) and the Mg-Sc-Sr extract (5020.2 ± 460.9) after 4 h of incubation was significantly higher compared to the control group (1758.0 ± 576.0) (*p* < 0.05). Figure 5D–F,J show tube formation after 24 h incubation of HUVECs with control, Mg, and Mg-Sc-Sr extract. The total tube length for Mg-Sc-Sr (10,413.6 ± 1014.6 μm) obtained significantly higher than the control (4723.2 ± 328.5 μm) and Mg (5759.6 ± 429.6 μm) (*p* < 0.05). A similar trend was obtained in HUVECs incubated with the control, Mg, and Mg-Sc-Sr extract after 48 h of incubation (Figure 5G–J). After 48 h, the measured total tube length of HUVECs cultured with Mg-Sc-Sr (10,352.8 ± 1140.0 μm) was significantly higher than the total tube length of HUVECs cultured with Mg (5988.2 ± 669.7 μm) and control (4698.4 ± 507.6 μm) that indicates the potential of Mg-Sc-Sr extract to enhance tube formation. In the control, the Mg and Mg-Sc-Sr groups, the total tube length after 24 h and 48 h of incubation was significantly higher than the total tube length at 4 h (*p* < 0.05). However, at 24 h and 48 h of incubation, the differences in total tube length between the Mg extracts and control were insignificant (*p* > 0.05).

The number of HUVECs meshes at 4 h of HUVECs culture with Mg extract (5.8 ± 0.8), and Mg-Sc-Sr extract (8.8 ± 0.8) was significantly higher compared to the control group (1.0 ± 1.0) (Figure 5K). After 24 h, the number of meshes for HUVECs cultured with Mg-Sc-Sr extract (16.0 ± 1.2) was significantly higher than the control group (7.2 ± 0.8) and Mg (11.6 ± 1.7). In addition, the number of meshes for HUVECs treated with Mg-Sc-Sr extract was significantly higher than HUVECs cultured with Mg extract. After 48 h of incubation, the number of meshes quantified for HUVECs cultured in Mg-Sc-Sr extract (21.8 ± 5.4) was significantly higher than the control group (11.2 ± 1.3) and Mg (12.0 ± 2.0). No significant difference was obtained for the number of meshes in Mg and control groups at 48 h. In the Mg-Sc-Sr group, the number of meshes at 48 h was significantly higher than the number of meshes at 24 h and 4 h (*p* < 0.05). 

**Figure 6:**
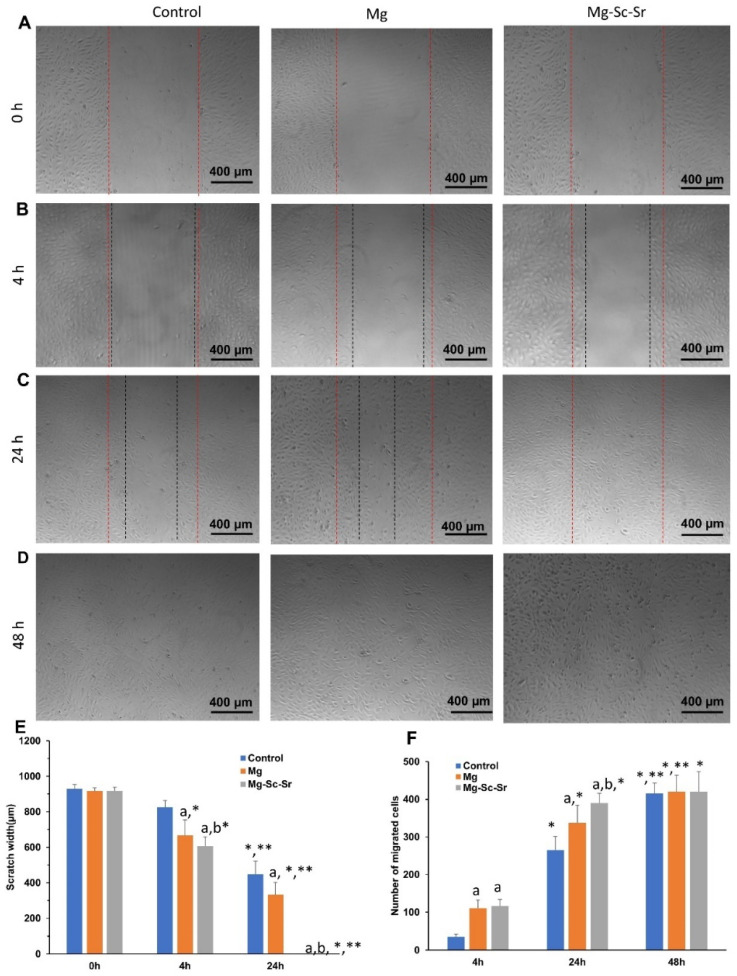
Scratch assay of HUVECs cultured with the control, Mg, and Mg-Sc-Sr extractions in vitro. (**A**) Images of initial wound scratch. Representative migration images of HUVECs after culturing with the control group, Mg extract, and Mg-Sc-Sr extract after (**B**) 4 h, (**C**) 24 h, (**D**) 48 h, (**E**) quantification of scratch width, (**F**) Quantification of the number of migrated cells. The width between red dots shows the primary scratch and the width between black dots shows the scratch width after incubation with or without extracts. Significant differences based on two-way ANOVA: a: *p* < 0.05 Mg and Mg-Sc-Sr vs. control, b: *p* < 0.05 Mg-Sc-Sr vs. Mg. For scratch width: *: *p* < 0.05 4 h and 24 h vs. 0 h, **: *p* < 0.05 24 h vs. 4 h for each experimental group. For the number of migrated cells: *: *p* < 0.05 24 h and 48 h vs. 4 h, **: *p* < 0.05 48 h vs. 24 h for each experimental group.

To evaluate the migration potential and wound-healing ability of HUVECs cultured with the control, Mg, and Mg-Sc-Sr extracts, a wound-healing (scratch) assay was performed. As indicated in Figure 6A–E, the scratch width in the samples cultured with Mg-Sc-Sr extract (605.6 ± 52.5 μm) and Mg (666.4 ± 88.2) was significantly lower than the control (826.4 ± 36.4 μm) after 4 h of incubation. In addition, the scratch width obtained for the Mg-Sc-Sr-treated group was significantly lower than that of the Mg-treated group. The ‘wounds’ were almost fully recovered in the Mg-Sc-Sr extract-treated HUVEC at 24 h. The calculated scratch width in HUVECs cultured with Mg-Sc-Sr and Mg (332.8 ± 68.2 μm) was higher than in the control group (448.6 ± 73.2 μm) groups, indicating a faster wound closure. In addition, the wound size in the Mg-Sc-Sr group was significantly reduced compared to the Mg group. No scratches were found in the control group, Mg, and Mg-Sc-Sr after 48 h of incubation (Figure 6D). The scratch size for the Mg-Sc-Sr-treated group was significantly reduced after 24 h of incubation compared to the Mg-Sc-Sr-treated group for 4 h.

As presented in Figure 6F, the numbers of migrated HUVECs for samples cultured with Mg-Sc-Sr extract (115.8 ± 18.4) and Mg extract (110.8 ± 21.4) were significantly higher than the control values (36.0 ± 7.2) after 4 h of incubation. No significant difference was obtained between Mg and Mg-Sc-Sr. After 24 h, the number of migrated HUVECs for cells cultured with Mg-Sc-Sr (390.0 ± 25.9) and Mg (337.2 ± 47.2) obtained significantly higher than the control (265.1 ± 36.4). In addition, the number of migrated cells for the Mg-Sc-Sr-treated group was significantly higher compared to the Mg-treated group. After 48 h, no significant difference obtained between experimental groups (*p* > 0.05), and the wound was fully recovered. The number of migrated HUVECs in the Mg-Sc-Sr group was significantly higher after 24 h and 48 h of incubation compared to 4 h, but the number of migrated cells between 24 h and 48 h was not significant. Data from HUVECs treated with Mg-Sc-Sr extracts show improvement in cell migration ability. 

### 3.3. Osteogenesis

**Figure 7:**
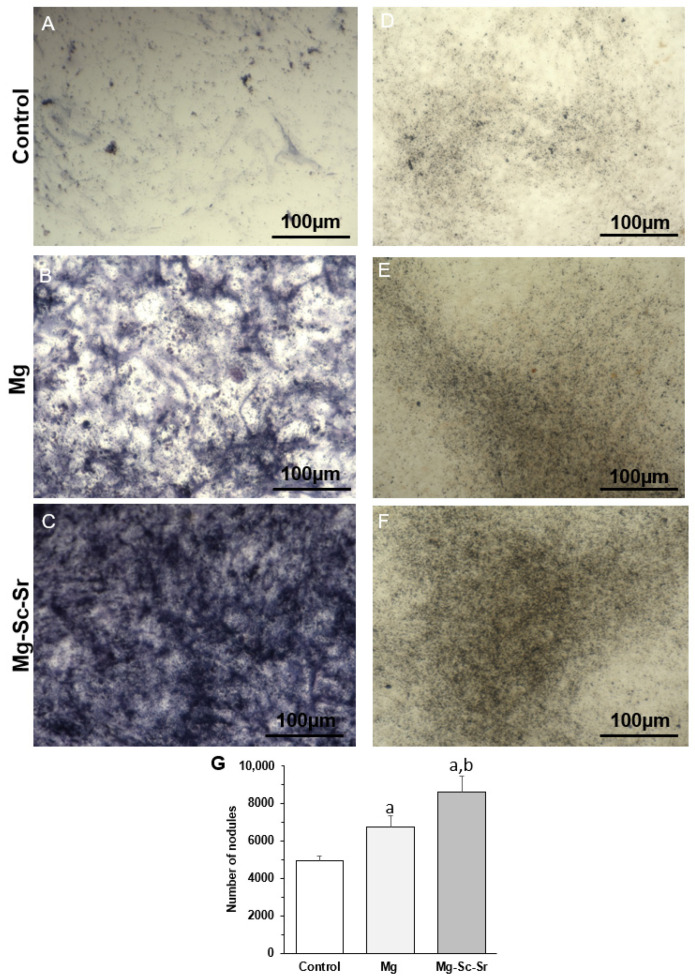
In vitro assay of osteogenic differentiation of hBM-MSCs and HUVECs cultured with alloy extracts: ALP staining of hBM-MSCs and HUVECs cultured with (**A**) control (differentiation medium without disc extract), (**B**) Mg disc extract, (**C**) Mg-Sc-Sr disc extract for seven days. A dark blue stain indicates more ALP activity by sample. Von Kossa staining of hBM-MSCs and HUVECs cultured with (**D**) Control (differentiation medium without disc extract), (**E**) Mg extract, (**F**) Mg-Sc-Sr disc extract for 21 days. More black precipitation indicates higher Ca deposition in the sample. (**G**) Quantitative analysis of Von Kossa staining, significant differences: a: *p* < 0.05 Mg and Mg-Sc-Sr vs. control, b: *p* < 0.05 Mg-Sc-Sr vs. Mg.

ALP and Von Kossa staining were used to identify osteogenic properties (Figure 7A–G). Figure 7A–C showed that the ALP staining on day 7 indicated higher ALP expression in hBM-MSC-HUVECs incubated with Mg-Sc-Sr extract than in cells incubated with Mg extract or the control group. ALP expression in hBM-MSCs-HUVECs was also higher in cells incubated with Mg extract compared to ALP stain intensity in the control group. The ALP staining result in cells incubated with Mg-Sc-Sr extract showed that this group had the highest ALP activity.

To examine the calcium deposition, hBM-MSC and HUVECs were cultured with the control, Mg, and Mg-Sc-Sr in differentiation extracts for 21 days. The ability of the disc extracts to promote bone formation could be directly correlated to the intensity of von Kossa staining. Figure 7D–G indicated higher Von Kossa staining (calcified nodules stained black) in cells cultured with Mg-Sc-Sr disc extracts compared to the cells cultured alone or with Mg disc extracts that included lower black stained nodes. As shown in Figure 7G, the quantified number of calcified nodules that stained black confirmed a significant increase (*p* < 0.05) in the mineral deposition by cells cultured with Mg-Sc-Sr extract (8562.21 ± 853.54) and Mg extract (6756.60 ± 567.50), compared to control (4938.02 ± 231.51). In addition, the number of nodules in cells cultured with Mg-Sc-Sr extract was significantly higher than in cells cultured with Mg (*p* < 0.05). As shown in the results, Mg-Sc-Sr extract promoted calcium deposition and improved osteogenic properties. 

## 4. Discussion 

We developed an Mg-Sc-Sr alloy that simultaneously releases Mg^2+^ and Sr^2+^ to enhance new bone formation and new vessel formation. This will further facilitate the application of Mg alloys in osteogenic fixation. In this study, we evaluated the effects of adding Sc and Sr elements to Mg matrices on microstructure, angiogenic character, and osteogenic properties. Herein, 2 wt.% Sr was used to enhance the angiogenesis and osteogenesis of the Mg alloy. Previous studies reported the addition of 0–2 wt.% Sr reduces the material corrosion rate and increases the corrosion uniformity in Sr-containing alloys [31,35,43,44]. Therefore, 2 wt.% Sr was added to maintain corrosion uniformity and maximize angiogenic and osteogenic properties. Many alloying elements have been used to limit the high corrosion of Mg [12]. However, avoiding elements such as Be, Nb, Cr, Ni, and Co is crucial to avoid long-term systemic inflammatory reactions [11]. Therefore, 2 wt.% Sc was added to increase the corrosion resistance without noticeable toxicity [45]. The effects of Sr and Sc addition on microstructure are discussed in the next paragraph.

The XRD pattern of Mg indicated the dominant α-Mg phase peaks. In the Mg-Sc-Sr alloy, the Mg_17_Sr_2_ intermetallic phase and α-Mg phase were observed. Based on the limited solubility of Sr (0.11%) in Mg, the Mg_17_Sr_2_ intermetallic phase forms in Sr-containing alloys [2]. The Mg_17_Sr_2_ formation refines the Mg grains via a grain-growth restriction mechanism during the solidification stages in the casting process [31,41,42]. As mentioned earlier, the elemental Mg and Sr can improve osteogenesis and angiogenesis. The combination of them (Mg_17_Sr_2_ intermetallic phase) may also be the reason for the increased osteogenesis and angiogenesis. The lower degradation rate of Mg_17_Sr_2_ could cause a longer systematic release of Mg and Sr from the Mg-Sc-Sr alloy. In addition, the structural grain refinement in alloys can change cellular differentiation, cell adhesion, and gene expression [46]. In our study, we performed indirect cell culture using alloy extracts. In future studies, direct cell culture experiments can be performed to evaluate the grain refinement effect of Mg-Sc-Sr alloy on the osteogenic differentiation of cells, tube formation, and scratch closure. 

Moreover, the chemical composition of the surface layers for Mg and Mg-Sc-Sr discs showed the existence of Mg in the elemental metallic form (Mg) and oxidized state (MgO) in XPS measurements. The MgO layer can prolong the implant’s life span and work as a protective barrier on the implant’s surface to increase corrosion resistance [47,48]. This MgO layer on the surface of Mg-Sc-Sr could be a potential reason for reducing the corrosion rate by adding a protective layer on the surface of discs [49,50,51]. However, other studies found that increasing the MgO content can have an adverse effect on corrosion resistance [47,48] because the hydrophilic behavior of MgO increases the wetting ability of the surface by water and exposes the Mg-Sc-Sr implant to water for an extended period, resulting in the reduction of corrosion resistance [52]. 

Our results showed a lower amount of MgO on the surface of Mg-Sc-Sr than Mg. This low amount of MgO on the surface of Mg-Sc-Sr could be a potential reason for improving the corrosion resistance by adding a protective and uniform layer on the surface of the discs [49,50,51]. In addition, during solidification, MgO can promote heterogeneous nucleation and increase grain refinement, which improves corrosion resistance. The formation of MgO can upregulate angiogenesis and osteogenesis-related gene expressions, such as VEGF, BMP2, and RUNX2, that enhance the differentiation of stem cells to osteoblasts [52]. In the following discussion, the effect of Sr on the osteogenic properties of Mg is discussed. 

Mg can enhance extracellular matrix mineralization, ALP activity, and osteogenesis-related gene expression, indicating improved osteogenesis character [9,53,54,55,56,57]. The ALP activity assessment and Von Kossa staining were performed to verify the osteogenic property of developed Mg-Sc-Sr. Significantly higher ALP staining of Mg-Sc-Sr compared to Mg and the control group after seven days of incubating HUVECs and hBM-MSCs confirmed the effect of Sr on Mg to significantly enhance the ALP activity and osteogenesis, which is consistent with the previous studies on Sr-containing alloys [58,59,60,61]. Our Von Kossa staining results also showed a higher amount of deposited calcium nodes in cells incubated with Mg-Sc-Sr extract, indicating a high potential for new bone formation. The possible molecular mechanisms underlying osteogenesis promotion by Mg are activation of mitogen-activated protein kinases/extracellular signal-regulated kinases (MAPK/ERK) signaling pathway and/or Wnt signaling pathways leading to an increase in osteogenic differentiation of stem cells to osteoblasts [62,63]. Additionally, the Sr-containing alloys showed upregulation of osteogenic-related gene expression, including Osterix (OSX), Runt-related transcription factor 2 (Runx2), Osteopontin (OPN), Osteocalcin (OCN), and Collagen I (COL I) [64,65,66]. The Sr addition can enhance the release of prostaglandin E-2 through cyclooxygenase 2 (COX2) expression and thereby increase osteoblastic differentiation of hBM-MSCs [67]. In addition, additional molecular pathways, such as the fibroblast growth factor/fibroblast growth factor receptor (FGF/FGFR) signaling pathway, have been indicated to be involved in strontium-mediated osteogenesis [68]. To accelerate the osteogenesis of a bone implant, improving the angiogenesis in addition to osteogenesis is vital [9,19]. The effect of Sr on angiogenesis potentiation by Mg alloys is discussed in the following discussion.

Angiogenic properties are necessary to repair large bone defects because new vessel formation can improve the transport of required nutrients and oxygen for osteogenesis at the fracture site and facilitate the transfer of cellular waste [25,69]. Higher angiogenic properties, such as upregulated related protein expression, endothelial tube formation, cell migration, and neovascularization in bone defect models, are reported for Sr-incorporated alloys [27,28]. Here, we measured VEGF expression as an essential regulator of angiogenesis that increases the proliferation and migration of HUVECs and plays an indirect role in osteogenesis by promoting angiogenesis [70,71,72]. The upregulation of VEGF in Mg-Sc-Sr extract-treated HUVECs confirmed our alloy’s impact on early angiogenesis. Then, scratch tests were performed to prove the critical effect of HUVECs migration in enhancing angiogenic character. The potential of HUVECs migration was assessed as the number of migrated HUVECs and the scratch width at each time point. As results showed, the number of migrated HUVECs was significantly higher for cells cultured with Mg-Sc-Sr extract than cells cultured alone or with Mg. The increased cell number was additionally reflected in the rapid decrease in scratch width and the extent of wound closure. The scratch was fully closed for Mg-Sc-Sr-treated HUVECs after 24 h of incubation. In contrast, scratches treated with Mg extract were not completely closed until 48 h of incubation.

Additionally, tube formation quantification was performed as an in vitro descriptor of tubulogenic ability. The significant increase in the total number of tubes and mesh numbers showed the tubulogenic ability of Mg-Sc-Sr extract. Our results in the angiogenic evaluations were consistent with previous results. Although the angiogenesis mechanism of Sr is not clear, most of the studies showed that Sr enhances the expression of angiogenic factors such as vascular endothelial growth factor (VEGF), fibroblast growth factor (bFGF), and hypoxia-induced factor α (HIF-α) that enhance angiogenesis [27,28].

Thus, our study showed that Mg-Sc-Sr is a promising Mg alloy for bone repair applications. The Mg-Sc-Sr alloy can be used for bone defect restoration, such as pins, screws, and tissue engineering scaffolds, by showing improved osteogenic and angiogenic properties in vitro. The Mg-Sc-Sr alloy could enhance bone formation and early vascularization in vitro. In the future, FESEM assessment, wetting properties, scratch properties, and degradation rate measurement in vitro and in vivo experiments will be required on Mg-Sc-Sr to obtain its potential as an implant in the human body. In addition, assessing the effect of bioactive coatings, such as calcium silicate [73] or adding MgO nanoparticles [74], on the surface of the Mg-Sc-Sr alloy could be beneficial. 

## 5. Conclusions

The Mg and Mg-Sc-Sr alloy were comprehensively investigated to reveal their microstructural refinement, angiogenesis, and osteogenesis. The XRD pattern showed α-Mg phase for Mg and α-Mg and Mg_17_Sr_2_ intermetallic phases for Mg-Sc-Sr alloy. The metallography results showed the grain size for Mg-Sc-Sr significantly decreased (76.84%) compared to Mg. A significant osteogenic differentiation of hBM-MSCs co-cultured with HUVECs cultured with Mg-Sc-Sr extract was obtained by ALP staining and the number of nodules in Von Kossa staining (73% increase) compared to the control. Moreover, the HUVECs cultured with Mg-Sc-Sr extracts demonstrated enhanced angiogenesis measured by the number of migrated cells (1.5-fold increase) than in the control after 24 h of incubation. Also, the scratch width was reduced significantly for the HUVECs cultured with Mg-Sc-Sr extracts compared to the control, and the scratch completely closed after 24 h of incubation. We concluded that the total tube length (1.2 increase) and the number of formed meshes (0.9 increase) were enhanced for the HUVECs cultured with the Mg-Sc-Sr extracts than those in the control group after 48 h of incubation.

## Figures and Tables

**Table 1:** The chemical compositions of Mg and Mg-Sc-Sr alloys.

Element Concentration (%)	Mg	Sc	Sr	P	Cl	Ca	Si	Zn	Cr	Ti	Mn	Fe
Mg	98.4340	-	-	0.7580	0.4790	0.2270	0.0294	0.0048	0.0158	-	0.0322	0.0188
Mg-Sc-Sr	94.120	1.369	2.003	1.223	0.657	0.488	-	-	0.0074	0.0058	0.0824	0.0355

## Data Availability

The corresponding author will make the data available upon reasonable request.
